# The effects of ulinastatin in multiple organ failure: a retrospective observational study in a single center ICU

**DOI:** 10.1186/2197-425X-3-S1-A878

**Published:** 2015-10-01

**Authors:** M Uchida, T Abe, K Ono

**Affiliations:** Department of Emergency and Critical Care Medicine, Dokkyo Medical University, Shimotsuga-gun Mibu-machi, Japan; Tsukuba Medical Center Hospital, Department of Emergency and Critical Care Medicine, Tukuba-shi, Japan

## Introduction

Ulinastatin, an urinary protease inhibitor, might reduce mortality of patients with systemic inflammatory response syndrome such as ARDS [1] and sepsis [2].

## Objectives

Our aim was to evaluate the effectiveness of ulinastatin on 28-day mortality among patients with multiple organ failure.

## Methods

We conducted a retrospective observational study of patients who were diagnosed multiple organ failure within 24 hours from admission in a general ICU of a tertiary care hospital in Japan. The study duration was from January 2009 to December 2012. The primary outcome was 28-day all cause mortality. The intervention was received ulinastatin for three days and more. Patients were also stratified by category of diseases as subgroup analysis.

## Results

A total of 212 patients with multiple organ failure met inclusion criteria during this study period. At baseline, mean age was 66.2 ± 14.7 years old, male was 146/212 (69%), mean APACHE II score was 24.7 ± 7.9, 206/212 (97 %) patients received mechanical ventilation, and 205/212 (96 %) patients were on vasopressor. Overall, 79/212 (37%) patients received ulinastatin. 28-day all cause mortality was 43/212 (20%). There were no significant differences between ulinasatin group and control group in age, gender, APACHE II score. Ulinastatin group had higher prevalence of sepsis (35/79 (44.3%) vs. 29/133 (21.8%), *P* = 0.001). They were more likely to receive corticosteroid, vasopressor, renal replacement therapy, and veno-arterial extra-corporeal membranous oxygenation (VA-ECMO). The mortality was not significantly different between the ulinastatin group and control group (20/79 (25.3%) vs. 23/133 (17.3%), *P* = 0.163). In logistic regression after adjusting for APACHE II score, there was not significant difference in the mortality (OR = 1.59; 95% CI, 0.79 - 3.21). The result was similar after adjusting for APACHE II score, sepsis, respiratory disorder, therapeutic interventions including corticosteroid, vasopressor, renal replacement therapy, and VA-ECMO. (OR = 1.29; 95% CI, 0.56 - 2.99). In sepsis patients, ulinastatin did not reduce the mortality, either. (OR = 1.92; 95% CI, 0.52 - 7.13).

## Conclusions

Ulinastatin was not associated with survival in patients with multiple organ failure.
